# A Collection on the prevention, diagnosis, and treatment of sexually transmitted infections: Call for research papers

**DOI:** 10.1371/journal.pmed.1002333

**Published:** 2017-06-27

**Authors:** Nicola Low, Nathalie Broutet, Richard Turner

**Affiliations:** 1 Institute of Social and Preventive Medicine, University of Bern, Bern, Switzerland; 2 World Health Organization, Geneva, Switzerland; 3 PLOS Medicine, Public Library of Science, Cambridge, United Kingdom

## Abstract

Nicola Low and colleagues announce a call for research papers on sexually transmitted infections, to accompany a Collection on the topic.

Sexually transmitted infections (STIs) are common, diverse, and dangerous to health—extending from bacterial diseases that may be readily treatable once diagnosed to viral infections such as HIV that can be life-threatening and, as yet, have no cure. A wide range of sexually transmissible pathogens have adverse effects on sexual and reproductive health, including infertility in women and several different types of cancer, with the global burden of cervical cancer a particular concern. Having an STI can also lead to low self-esteem, stigma, and sexual dysfunction. Moreover, some STIs are transmitted from mother to child and thereby lead to poor pregnancy, neonatal, and child health outcomes, including stillbirth. Emerging pathogens that prove to be sexually transmissible, recently exemplified by Ebola and Zika viruses, can be expected to evoke substantial and widespread concern where the risks and possible consequences of a disease outbreak are sketchily understood.

According to WHO [1], more than 30 different bacteria, viruses, and parasites lead to greater than 1 million sexually transmitted infections each day. Chlamydia (with an estimated 131 million new infections annually), gonorrhea (78 million infections), syphilis (5.6 million infections), and trichomoniasis (143 million infections) are 4 of the most common infections worldwide that can, at present, be treated with existing antibiotic regimens. However, antimicrobial resistance is a growing threat, particularly for gonorrhea and *Mycoplasma genitalium*. The most prevalent viral STIs are genital herpes simplex virus infection (affecting an estimated 500 million people worldwide) and human papillomavirus (HPV) infection (affecting 290 million women and leading to some 500,000 cases of cervical cancer annually). While antiviral treatment may control recurring herpes in some people, disease prevention and, where possible, vaccine development and deployment are priorities in the absence of curative interventions. Enmeshed as they are in human biology, behavior, and culture, STIs provide great challenges to those responsible for disease surveillance, planning services, and provision of treatment in all countries.

Because of the enormous burden of STIs and their wide-ranging adverse health effects, decisive action will be an essential part of efforts to meet the health component of the Sustainable Development Goals, and the targets of the global health sector strategy on sexually transmitted infections 2016–2021 [2], which were adopted by the 69th World Health Assembly in May 2016. Accompanying this Editorial are several articles forming part of a WHO-sponsored Collection addressing global policy and practice aimed at achieving control of STIs. Andrew Seale and colleagues discuss the development process for the global strategy to counter STIs [3]. Global targets for STI control specify, by 2030, achievement of a 90% reduction in syphilis incidence; a 90% reduction in gonorrhea incidence; and occurrence of 50 or fewer cases of congenital syphilis per 100,000 live births in 80% of countries, and Melanie Taylor and colleagues discuss systems for STI surveillance and monitoring of treatment resistance towards these targets [4]. Taylor and colleagues also discuss programs and criteria aimed at elimination of mother-to-child transmission of syphilis and HIV [5]. Finally, Paul Bloem and colleagues discuss HPV vaccination for control of cervical cancer and other HPV-related diseases [6] in different settings, illustrating the prospects of new interventions for STI control. Further discussion articles will appear in future issues of *PLOS Medicine*, and, as part of the cross-journal Collection, research papers are being published in *PLOS Medicine* and in other PLOS journals [7].

To accompany this Collection, we are inviting submission of reports of high-quality research studies with the potential to inform clinical practice or thinking relevant to STIs, focused on the following:

- Epidemiological studies on the incidence, prevalence, and disease burden of STIs, including emerging and re-emerging infections, mother-to-child transmission of STIs, and STIs in the context of new HIV prevention approaches;
- Molecular and genomic studies relevant to clinical advances in STI research, including antimicrobial resistance and microbiome analysis;
- Studies investigating treatment, partner notification, vaccination, behavioral, and combination interventions for STIs, with reports of randomized controlled trials particularly welcome;
- Implementation research, especially focused on interventions for STI prevention and point-of-care approaches to disease diagnosis in low- and middle-income countries, including qualitative research on issues such as stigma;
- Modelling and cost-effectiveness studies relevant to STIs, addressing prevention and treatment interventions.

Please submit your manuscript at http://journals.plos.org/plosmedicine/s/submit-now. We (NL and NB) will be the guest editors for the Collection, and successful submissions will be published, following peer review, from December 2017 onwards. Papers to be published in December should be submitted by **August 11, 2017** but submissions will still be considered, and can be included in the Collection, after that date. Presubmission inquiries are not required, but we ask that you indicate your interest in this call for papers in your cover letter.

## Abbreviations

HPV: human papillomavirus
STI: sexually transmitted infection
